# Trypanocidal Action of (−)-Elatol Involves an Oxidative Stress Triggered by Mitochondria Dysfunction

**DOI:** 10.3390/md10081631

**Published:** 2012-08-03

**Authors:** Vânia Cristina Desoti, Danielle Lazarin-Bidóia, Daniela Bueno Sudatti, Renato Crespo Pereira, Antonio Alonso, Tania Ueda-Nakamura, Benedito Prado Dias Filho, Celso Vataru Nakamura, Sueli de Oliveira Silva

**Affiliations:** 1 Postgraduate Program in Pharmaceutical Sciences, State University of Maringa, Colombo Avenue 5790, CEP 87020-900, Maringa, Parana, Brazil; Email: vaniadesoti@gmail.com (V.C.D.); dani.lazarin@bol.com.br (D.L.-B.); tunakamura@uem.br (T.U.-N.); bpdfilho@uem.br (B.P.D.F.); cvnakamura@gmail.com (C.V.N.); 2 Department of Marine Biology, Federal Fluminense University, PO Box 100644, CEP 24001-970, Niteroi, Rio de Janeiro, Brazil; Email: dbsudatti@gmail.com (D.B.S.); egbrecp@vm.uff.br (R.C.P.); 3 Institute of Physics, Federal University of Goias, CEP 74001-970, Goiania, Goias, Brazil; Email: alonso@if.ufg.br; 4 Department of Basic Health Sciences, State University of Maringa, Colombo Avenue 5790, CEP 87020-900, Maringa, Parana, Brazil

**Keywords:** (−)-elatol, *Trypanosoma cruzi*, Chagas’ disease, mitochondria, oxidative stress

## Abstract

Natural compounds have shown good potential for the discovery of new chemotherapeutics for the treatment of Chagas’ disease. Recently, our group reported the effective trypanocidal activity of (−)-elatol, extracted from the red macroalgae *Laurencia dendroidea* present in the Brazilian coast against *Trypanosoma cruzi*. However, the mechanism of action of this compound has remained unclear. There are only hypotheses concerning its action on mitochondrial function. Here, we further investigated the mechanisms of action of (−)-elatol on trypomastigotes of *T. cruzi*. For this, we evaluated some biochemical alterations in trypomastigotes treated with (−)-elatol. Our results show that (−)-elatol induced depolarization of the mitochondrial membrane, an increase in the formation of mitochondrial superoxide anion and loss of cell membrane and DNA integrity. Additionally, (−)-elatol induced formation of autophagic vacuoles and a decrease in cell volume. All together, these results suggest that the trypanocidal action of (−)-elatol involves multiple events and mitochondria might be the initial target organelle. Our hypothesis is that the mitochondrial dysfunction leads to an increase of ROS production through the electron transport chain, which affects cell membrane and DNA integrity leading to different types of parasite death.

## 1. Introduction

More than one hundred years after the discovery of Chagas’ disease there are only two drugs available for the treatment of this infection. These two drugs, nifurtimox and benznidazole, have variable efficacy, especially in the chronic phase of the disease. Using these drugs cause serious toxic side effects as well as expose the patient to prolonged treatment. Estimates indicate that there are about 10 million cases of Chagas’ disease worldwide [1] and around 50,000 to 200,000 new infections occurring every year [2]. In addition, more than 10,000 deaths per year are due to Chagas’ disease [1].

**Figure 1:**
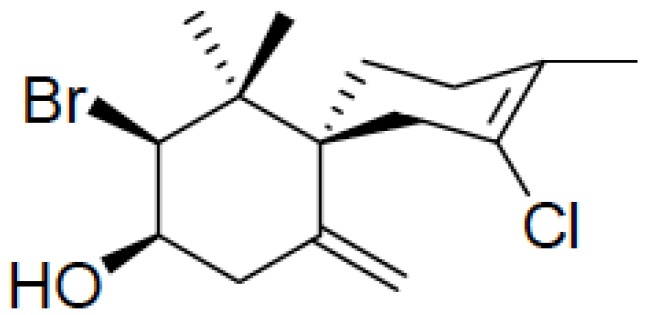
Chemical structure of (−)-elatol, the sesquiterpene extracted from red macroalgae *Laurencia dendroidea*.

In this context, the search for new effective and less toxic chemotherapeutic agents for the treatment of Chagas’ disease is increasing [3]. Although literature presents various studies regarding extracts and pure compounds obtained from plants and macroalgae with good potential for the treatment of this infection [4,5,6,7,8], little is known about their mechanisms of action. As an example, our group recently reported the effective trypanocidal activity of (−)-elatol (Figure 1), extracted from red macroalgae *Laurencia dendroidea*, present in the Brazilian coast on *Trypanosoma cruzi* (previously named elatol) [8]. However, nothing was described about the mechanism of action of this compound, only a hypothesis concerning its action on mitochondrial function [8]. In fact, the mitochondria of Trypanosomes exhibit unique characteristics that are distinct from mammalian mitochondria, making this organelle a major target of chemotherapeutic agents [9].

This prompted us to further investigate the involvement of mitochondrial dysfunction on *T. cruzi* death induced by (−)-elatol. This hypothesis is strongly based on our previous work, where transmission electron microscopy (TEM) data provided evidence of ultrastructural alterations such as swollen mitochondrial. Thus, we evaluated some biochemical alterations on trypomastigote forms treated with (−)-elatol in a way to better elucidate the relationship between mitochondrial dysfunction and the type of cell death triggered.

## 2. Results and Discussion

(−)-Elatol (Figure 1) has previously been reported to have trypanocidal [8], leishmanicidal [7], and antibacterial activity [10,11,12] and significantly active roles in ecological interactions, such as antiherbivore activity [13]. In the present study we focus our efforts on the trypanocidal activity of (−)-elatol in an attempt to delineate the putative mechanism of action of this compound. 

**Figure 2:**
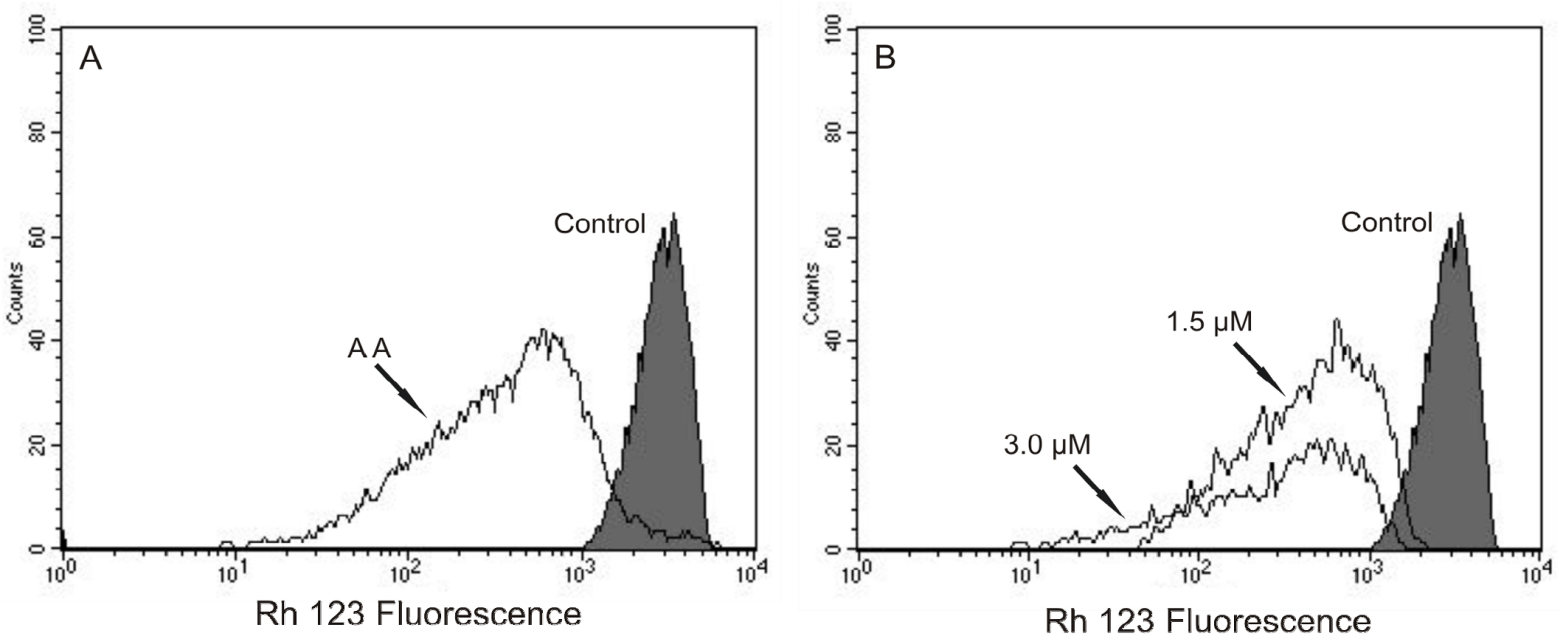
Flow cytometry analysis of trypomastigotes of *Trypanosoma cruzi* treated with (−)-elatol for 3 h and stained with Rh 123. (**A**) Trypomastigotes treated with 2.0 µM of antimicyn A (AA) (positive control); (**B**) Trypomastigotes treated with 1.5 and 3.0 µM of (−)-elatol for 3 h. Control group (untreated parasite) is also shown. Typical histograms of at least three independent experiments.

Based on our previous work that indicated, by electron microscopy, the effect of (−)-elatol on *T. cruzi* mitochondria and cell membranes [8], we decided to evaluate the mitochondrial membrane potential (ΔΨm) and the cell membrane integrity in (−)-elatol-treated trypomastigotes by flow cytometry. Histograms show a marked decrease in fluorescence intensity total rhodamine 123 (Rh 123), indicating mitochondrial depolarization in cells treated with 1.5 and 3.0 µM of (−)-elatol for 3 h, with ΔΨm reductions in a range of 80.0% (Figure 2B) when compared to the control group. A decrease in fluorescence intensity was also observed after 2 h of treatment, however the ΔΨm reductions were almost 3-fold smaller than those observed after 3 h (data not shown). The positive control antimicyn A (AA) induced 81.3% change in mitochondrial membrane potential (Figure 2A). 

In this context, our data adds further evidences that mitochondria are a target for (−)-elatol action, strengthening the idea introduced in our previous work [8]. In fact, increasingly well documented papers have described trypanocidal compounds targeting parasite mitochondrial function [9,14].

**Figure 3:**
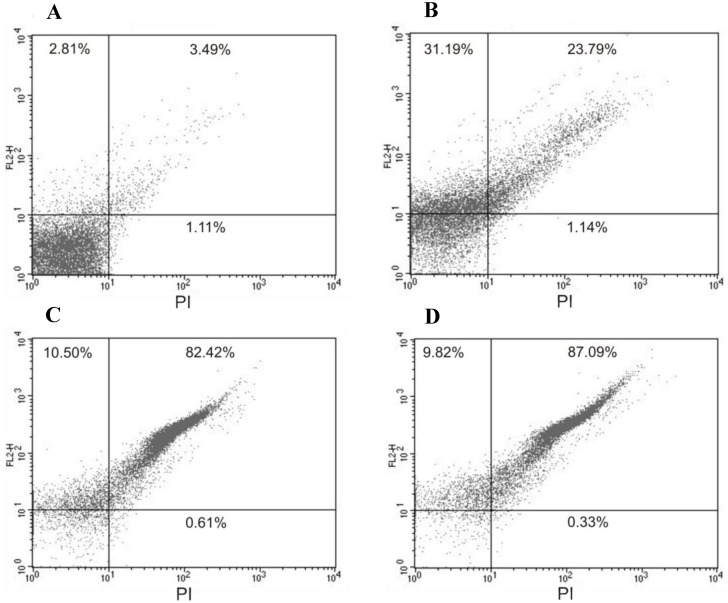
Flow cytometry analysis of trypomastigotes of *Trypanosoma cruzi* treated with (−)-elatol for 2 h and stained with propidium iodide (PI). (**A**) Control group (untreated cells);(**B**) Trypomastigotes treated with digitonin 40.0 µM (positive control); (**C**) Trypomastigotes treated with 1.5 µM (−)-elatol; (**D**) Trypomastigotes treated with 3.0 µM (−)-elatol. The numbers shows the percentage of PI-stained positive cells in upper right and left quadrant. Typical histograms of at least three independent experiments.

Our results also show that not only the mitochondria, a unique and essential organelle of trypomastigotes [15], was affected by (−)-elatol, but also the plasma membrane, a selective structure that controls the movement of substances in and out of cells essential for the maintenance of the parasite homeostasis. This effect was evidenced by propidium iodide (PI)-stained cells. Figure 3 shows an increase in the intensity of PI fluorescence in trypomastigotes treated with (−)-elatol at 1.5 and 3.0 µM for 2 h around 90.0% which is noticeably higher than the PI fluorescence of the control group, indicating alteration of cell membrane integrity. The positive control (B) with digitonin also shows an increase in fluorescence (of 55.0%). 

**Figure 4:**
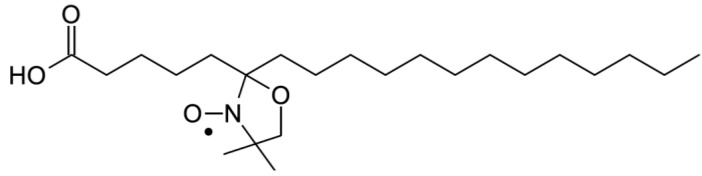
Chemical structure of spin label 5-doxyl stearic acid (5-DSA) used in this work.

**Figure 5:**
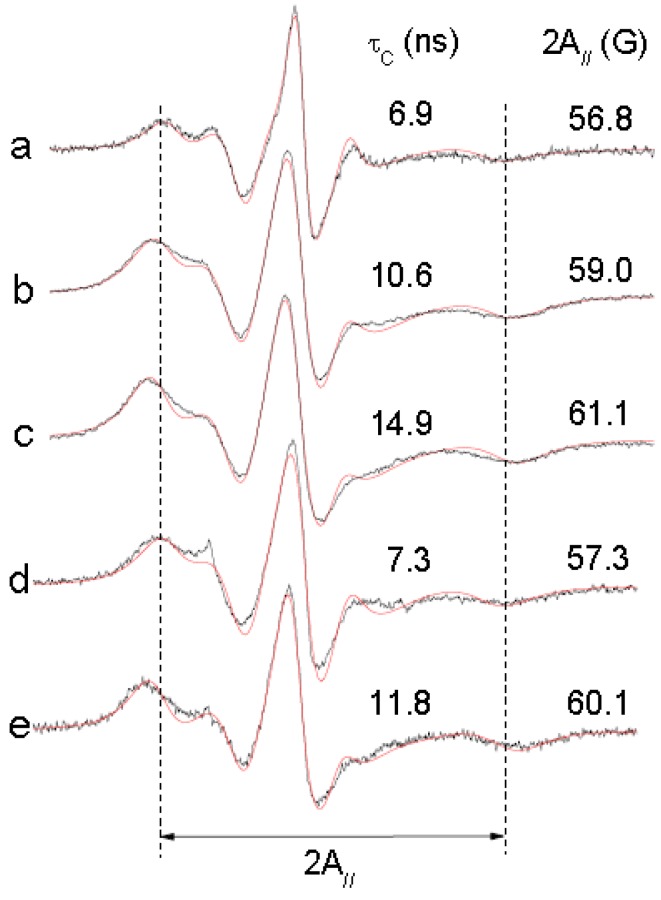
Experimental (black line) and best-fit (red line) electron paramagnetic resonance (EPR) spectra of spin label 5-DSA of trypomastigotes of *Trypanosoma cruzi* treated with (−)-elatol. The EPR spectra (**a**) and (**d**) were obtained from trypomastigotes without treatment (control samples); spectra (**b**) and (**e**) are from samples treated with 5.4 × 10^9^ (−)-elatol molecules/cell and the spectrum (**c**) is from a sample treated with 1.6 × 10^10^ (−)-elatol molecules/cell. EPR spectra were simulated with the fitting program NLLS and the values of the parameter rotational correlation time, τ_C_, obtained from the fit for each spectrum are indicated in nanosecond scale. The EPR parameter 2A_//_ is the separation in magnetic-field units between the first and last resonance lines (indicated by vertical lines) of the spectrum. The estimated experimental error for 2A_//_ and τ_C_ parameters are 0.5 G and 1.0 ns, respectively. Typical spectra of two independent experiments.

To confirm the effect of (−)-elatol on the cell membrane the experimental and best-fit electron paramagnetic resonance (EPR) spectra of spin label 5-doxyl stearic acid (5-DSA) (Figure 4) structured in the plasmatic membrane of trypomastigotes was made and are shown in Figure 5. These EPR spectra are typical for cellular membranes containing an appreciable amount of integral proteins. The treatment with (−)-elatol increased two EPR parameters, the outer hyperfine splitting, 2A_//_, and the rotational correlation time, τ_C_, indicating significant reduction in membrane lipid dynamics. 2A_//_ is a practice parameter measured directly in the EPR spectra (Figure 5). This has been widely used to monitor membrane fluidity even though, in principle, it is a static parameter associated with the orientation distribution of the spin labels in the membrane. 

The presence of the sesquiterpene (−)-elatol significantly increased the rigidity of the membrane of *T. cruzi* as evidenced by EPR spectra. The spin probe used in EPR is sparsely distributed in the membrane and, therefore, the spin probe spectroscopy only detects changes in membrane fluidity when a widespread change occurs.

All these cell membrane alterations induced by (−)-elatol could be a result of oxidative damage induced by reactive oxygen species (ROS) production. The increase of ROS can lead to destructive effects through the reaction with biological macromolecules such as lipids, proteins and DNA [16]. It is well known that mitochondria play an important role in the production of ROS through oxidative phosphorylation involving the electron transport chain. In certain situations, for example changes in mitochondrial membrane potential, an increase in the production of ROS through the electron transport chain is observed [9]. This increase contributes to mitochondrial damage followed by an increase of permeability of their membranes resulting in the release of apoptosis activating factors such as ROS toward the cytosol [17,18]. 

Thus, based on our results of cell membrane and mitochondrial membrane potential alterations we decided to evaluate the superoxide anion production (O_2_^•−^), by a very sensitive fluorimetric assay, in mitochondria of (−)-elatol-treated trypomastigotes. 

**Figure 6:**
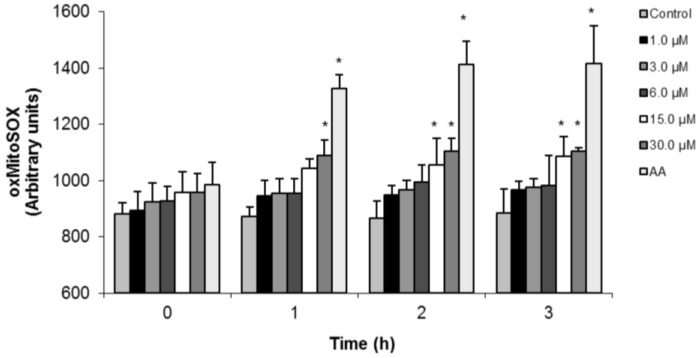
Mitochondrial O_2_^•−^ production in trypomastigote forms of *Trypanosoma cruzi* treated with (−)-elatol for up to 3 h. Mitochondrial O_2_^•−^ production was evaluated using the fluorescent probe MitoSOX. At the indicated times, parasites were used to fluorimetrically measure oxidized MitoSOX (oxMitoSOX). Results are expressed in arbitrary units as means ± SD of at least three independent experiments. Asterisks indicate significant differences relative to the control group (untreated cells) as identified by variance analysis (two-way) with Tukey post-test (*p* ≤ 0.05).

As shown in Figure 6, (−)-elatol induced an increase in the O_2_^•−^ production in all concentrations assayed starting from 1 h of incubation. However, 15.0 and 30.0 µM after 2 and 3 h were the most effective concentrations of (−)-elatol displaying a significant increase (about 20.0%) of mitochondrial O_2_^•−^ production when compared to the control group. The positive control with AA also induced an increase of mitochondrial O_2_^•−^ production (data not shown). 

**Figure 7:**
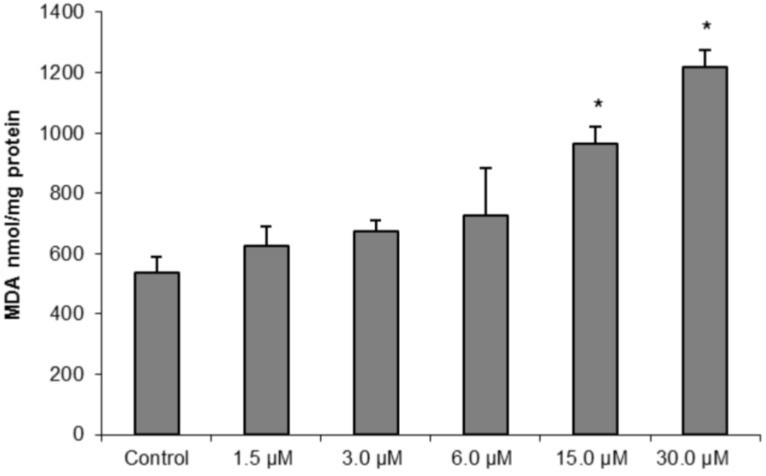
Determination of lipid peroxidation in trypomastigote forms of *Trypanosoma cruzi* treated with different concentrations of (−)-elatol for 3 h. The malondialdehyde (MDA) concentration was measured by thiobarbituric acid-reactive substances (TBARS) production. The results are expressed as means ± SD of at least three independent experiments. Asterisks indicate significant differences relative to the control group (untreated cells) as identified by variance analysis (one-way) with Tukey post-test (*p* ≤ 0.05).

To further confirm our hypothesis of oxidative damage induced by (−)-elatol-treated trypomastigotes, we measured the production of thiobarbituric acid-reactive substances (TBARS) (which is frequently used to quantify lipoperoxidation of the cell membrane and is expressed by the production of malondialdehyde (MDA)). The measurement of TBARS in trypomastigotes treated with 15.0 and 30.0 µM of (−)-elatol revealed a significant increase in lipid peroxidation after 3 h when compared to the control group (Figure 7). The lipid peroxidation data also gave more evidence of an increase in membrane rigidity of (−)-elatol-treated parasites. Lipid peroxidation alters essential structural components of cell membranes affecting cell membrane permeability and fluidity [19].

**Figure 8:**
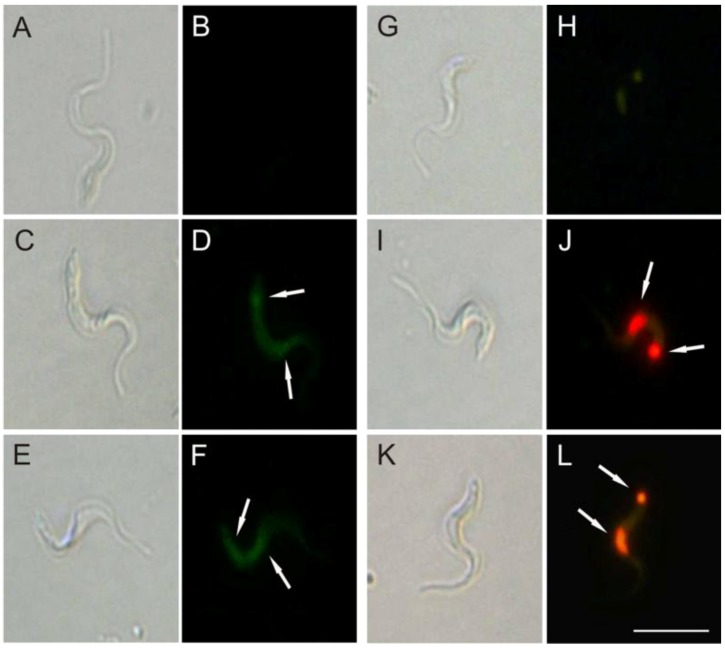
DNA fragmentation in trypomastigote forms of *Trypanosoma cruzi* treated with (−)-elatol for 24 h. TUNEL (panels **A**–**F**) and PI (panels **G**–**L**) were analyzed by fluorescence microscope. Gray column is differential interference contrast (DIC) and black column is fluorescence; (**A**,**B**,**G**,**H**) Representative images of untreated cells; (**C**,**D**,**I**,**J**) Representative images of trypomastigotes treated with 1.5 µM (−)-elatol; (**E**,**F**,**K**,**L**) Representative images of trypomastigotes treated with 3.0 µM (−)-elatol. Arrows indicate DNA fragmentation (green) and condensation and margination of chromatin (red). Bars: 10 µm.

The increase of O_2_^•−^ production induced by (−)-elatol might lead to a DNA break as well. As shown in Figure 8, bright fluorescence was observed in trypomastigotes treated with 1.5 and 3.0 µM of (−)-elatol for 24 h and staining with TUNEL (Figure 8D,F). Additionally, the counterstaining with PI (Figure 8J,L) denotes that (−)-elatol induced the condensation and margination of chromatin. The control without treatment was TUNEL and PI negative (Figure 8B,H). In addition, bright fluorescence was also observed with actinomycin D, a known apoptosis inducer (data not shown). 

In this context, and based on well-established literature, we can state that (−)-elatol induces an oxidative stress condition leading to cumulative oxidative damage in the parasite macromolecules. Besides lipid peroxidation, we showed that (−)-elatol-treated trypomastigotes can also trigger destructive effects on DNA evidenced by TUNEL and PI staining.

**Figure 9:**
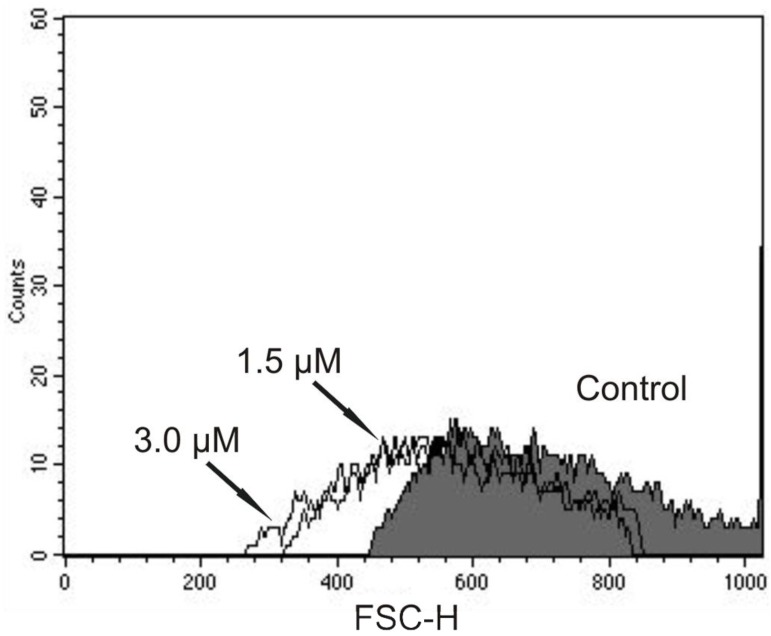
Flow cytometry analysis of trypomastigote forms of *Trypanosoma cruzi* treated with (−)-elatol for 24 h. Forward light scatter (FSC-H) was considered a function of cell size. Representative FACS histogram showing FSC-H of trypomastigotes treated with 1.5 µM and 3.0 µM (−)-elatol and the control group (untreated cells, gray full histogram). Typical histogram of at least three independent experiments.

The DNA fragmentation is one of the final steps in the apoptotic process and could be evidence of apoptosis in trypomastigotes treated with (−)-elatol. Therefore, we performed additional experiments to evaluate the cell shrinkage, a hallmark of apoptotic death as well. As shown in Figure 9, there was a decrease in cell volume in the presence of concentrations of 1.5 and 3.0 µM of (−)-elatol after 24 h, where reductions of 20.0% and 23.8% were observed, respectively. The positive control actinomycin D induced a decrease of 79.7% in the cell volume.

**Figure 10:**
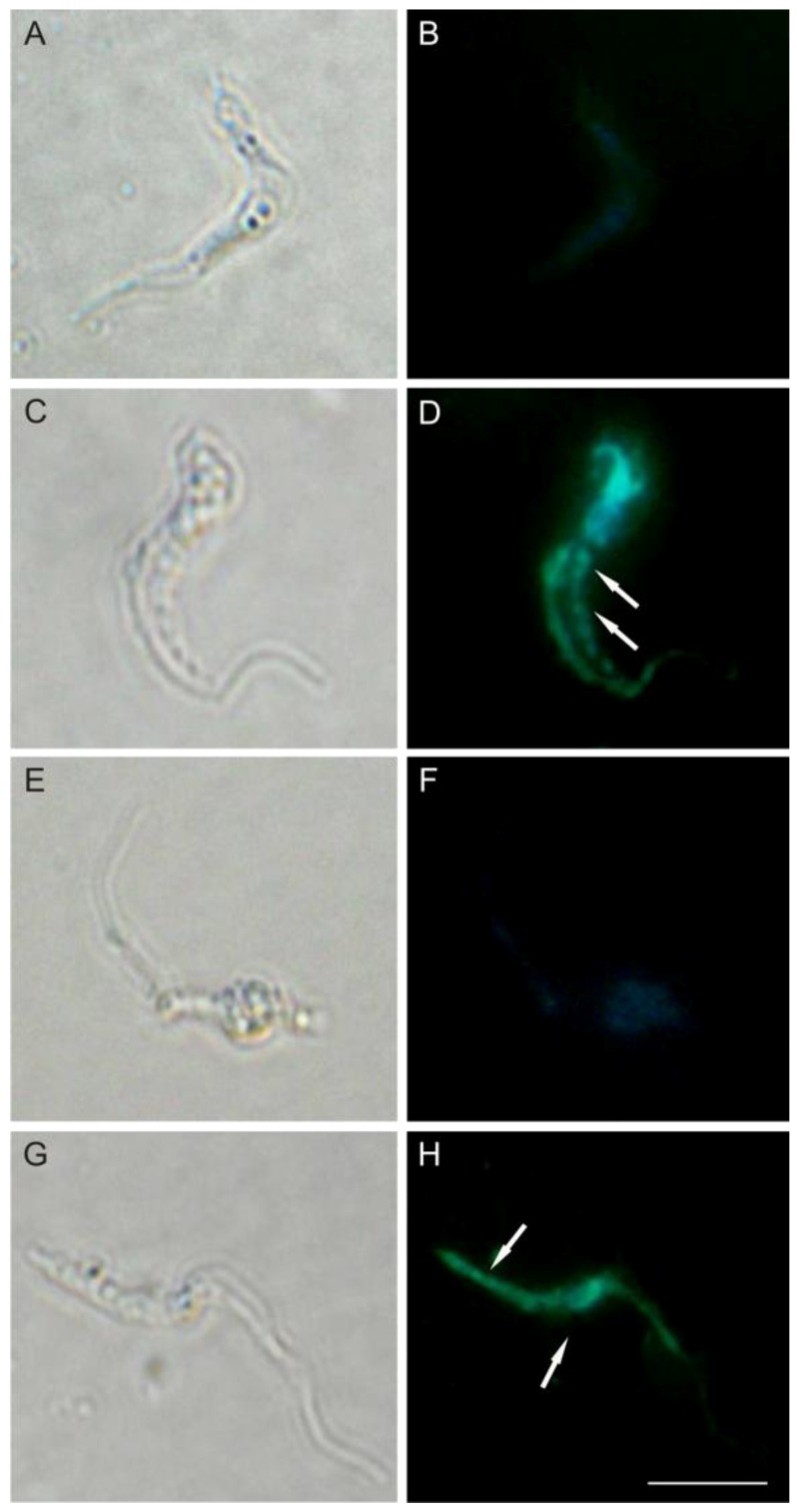
Determination of autophagy in trypomastigote forms of *Trypanosoma cruzi* treated with (−)-elatol for 24 h and stained with MDC. Gray column is DIC and black column is fluorescence. (**A**,**B**) Representative image of untreated cells; (**C**,**D**) Representativeimage of trypomastigotes treated with 1.5 µM (−)-elatol; (**E**,**F**) Representative image of trypomastigotes treated with 1.5 µM (−)-elatol + wortmannin; (**G**,**H**) Representative image of trypomastigotes treated with 3.0 µM (−)-elatol. Arrows indicate the stained autophagic structures. Bars: 10 µm.

Based on our previous work that showed by TEM the extensive cytoplasmic vacuolization on *T. cruzi* treated with (−)-elatol [8] we decided to evaluate if autophagy could also be a death pathway induced by (−)-elatol. For this, we evaluated autophagy by staining trypomastigotes treated with (−)-elatol with monodansylcadaverine (MDC), a fluorescent probe that accumulates in autophagic vacuoles [20]. As shown in Figure 10 the presence of fluorescence in rounded structures in cells treated with (−)-elatol revealed the formation of autophagic vacuoles (Figure 10D,H), unlike the untreated cells (Figure 10B). This effect could be partially prevented in trypomastigotes pre-treated with wortmannin (Figure 10F). 

Up to here our results indicate that (−)-elatol induced alterations that might be responsible for different types of cell death. For example, the alterations in mitochondria and the breakdown of the plasma membrane observed here and the distortion in the cell body described before [8] are all hallmarks of necrosis. The DNA fragmentation, one of the final steps in the apoptotic process, could be evidence of apoptosis in (−)-elatol-treated trypomastigotes. Additionally, the decrease in cell volume observed here in treated parasites is one more indicator of apoptosis [21]. Another type of cell death described for *T. cruzi* is autophagy, which is characterized by an increase in cytoplasmic vacuolization [22]. Our previous TEM data [8] point to the formation of cytoplasmic vacuoles in *T. cruzi* treated with (−)-elatol which suggest autophagic death. In this work we confirm autophagic death. This result is based on a fluorescent assay where the autophagic vacuoles were partially reduced by wortmannin, a PI3-K inhibitor, which is an enzyme of the signaling pathway involved in autophagy regulation [21,23].

## 3. Experimental Section

### 3.1. Chemicals and Materials

Actinomycin D, antimicyn A, bovine serum albumin, digitonin, dimethylsulfoxide, monodansylcadaverine, rhodamine 123, thiobarbituric acid, wortmannin and spin label 5-doxyl stearic acid, having the nitroxide radical moiety (doxyl) in the 5th carbon atom of the acyl chain, were purchased from Sigma-Aldrich (St. Louis, MO, USA); Dulbecco’s modified Eagle’s medium, fetal bovine serum was from Invitrogen (Grand Island, NY, USA); MitoSOX kit, propidium iodide, and TUNEL kit was from Invitrogen (Eugene, OR, USA) and protein assay kit was from Bio-Rad (Hercules, CA, USA). All other reagents were of analytical grade.

### 3.2. Isolation of (−)-Elatol from *Laurencia dendroidea*

(−)-Elatol was isolated from specimens of *L. dendroidea* collected by hand during low tide, in the midlittoral zone on the rocky coast of Cabo Frio Island (22°59′ S, 42°59′ W), Rio de Janeiro State, Brazil. The seaweed was stored in plastic bags and chilled on ice during transport to the laboratory. The specimens of *L. dendroidea* used in this study were identified by Dr. Mutue Toyota Fujii, and voucher specimens were deposited in the herbaria SP, Instituto de Botânica, São Paulo State, Brazil (SP number: 399789). *L. dendroidea* was dried in the dark at room temperature in order to avoid photolysis and thermal degradation. 

The air-dried algal material (300.0 g) giving 50 mg of (−)-elatol was successive and exhaustively extracted in *n*-hexane at room temperature for 15 days. The solvent was eliminated in a rotary evaporator, at low temperature (<50 °C), yielding 3.64 g of a dark green extract containing the sesquiterpene (−)-elatol, which was detected as a blue spot on TLC plates after spraying with a solution of ceric sulfate and sulfuric acid (2.1 g of Ce_2_(SO_4_)_3_·4H_2_O; 21 mL of H_2_SO_4_ and 300 mL of H_2_O), followed by heating at 100 °C for 3 min. An aliquot of HE (0.35 g) was submitted to preparative thin layer chromatography (PTLC) (Merck, silica gel 60 F_254_, 20 × 20 cm, mobile phase: *n*-hexane/ethyl acetate 8:2), to afford a yellowish oil (50 mg) which was identified as the sesquitepene (−)-elatol. The purity was confirmed by TLC (*R_f_* = 0.45), using *n*-hexane/AcOEt 8:2 as mobile phase, and by ^1^H-NMR spectroscopy (300 MHz), and comparison with the literature [24,25].

(−)-Elatol stock solutions (1 mg/mL) were prepared in dimethyl sulfoxide, stored at 4 °C. All groups (including controls) were tested at final concentrations of less than 1% dimethylsulfoxide (DMSO), a concentration found not to affect trypomastigotes (data not shown). The tested concentrations were based on effective concentration (EC_50_) about 1.5 µM [8].

### 3.3. Parasites and Cells Cultures

*T.* *cruzi* trypomastigote forms (Y strain) (95% of purity) were obtained from the supernatant of an infected LLCMK_2_ cells monolayer (epithelial cell of monkey kidney; *Macaca mulatta*) in DMEM medium supplemented with 2 mM L-glutamine, 10% heat-inactivated fetal bovine serum (FBS), 50 mg/L gentamicin, and buffered with sodium bicarbonate in a 5% CO_2_ air mixture at 37 °C. Sub-confluent cultures of LLCMK_2_ cells were infected with 5 × 10^6^ trypomastigotes. Extracellular parasites were removed after 24 h, the cells washed, and these cultures were maintained in DMEM medium containing 10% FBS, until trypomastigotes emerged from the infected cells.

### 3.4. Mitochondrial Membrane Potential and Cell Membrane Integrity Assays

Trypomastigotes (1 × 10^7^ cells/mL) treated or untreated with 1.5 and 3.0 µM of (−)-elatol, for 2 and 3 h at 37 °C, were washed and incubated with 5.0 µg/mL of Rh 123 for 15 min to evaluate the ΔΨm and 0.2 µg/mL of PI for 10 min to verify possible alteration in cell membrane integrity. The compound AA at a concentration of 2.0 µM was used as a positive control for measurement of mitochondrial membrane potential and digitonin at 40.0 µM for cell membrane integrity. Data acquisition and analysis were performed using a FACSCalibur flow cytometer (Becton-Dickinson, Rutherford, NJ, USA) equipped with the CellQuest software (Joseph Trotter, Scripps Research Institute, La Jolla, CA, USA). A total of 10,000 events were acquired in the region previously established as that corresponding to the parasites. Alterations in the fluorescence of Rh 123 were quantified as the percent of reduction of the fluorescence compared with the control (untreated parasites).

### 3.5. Spin Labeling

A small aliquot (3 µL) of stock solution of spin label 5-DSA in ethanol (2 mg/mL) was transferred to an eppendorf tube. After that, the solvent was evaporated and about 1 × 10^8^ trypomastigotes/mL, suspended in 30 µL of phosphate-buffered saline (PBS), was added on the film of spin label and gentle agitation applied. After spin labeling, 1 or 3 µL of a stock solution of (−)-elatol in ethanol (300 mg/mL) was applied to the cell suspension and gently mixed. The cells were then introduced into 1-mm i.d. capillary for EPR measurements, which were sealed by flame.

### 3.6. EPR Spectroscopy

EPR spectroscopy was performed with a Bruker ESP 300 spectrometer (Rheinstetten, Germany) equipped with an ER 4102 ST resonator. The instrument settings were: microwave power of 10 mW; modulation frequency of 100 KHz; modulation amplitude of 1.0 G; magnetic field scan of 100 G; sweep time of 168 s; and detector time constant of 41 ms. EPR spectra simulations were performed using the NLLS program (nonlinear least-squares fitting program) developed by Freed and coworkers [26]. In the spectral calculations, the NLLS program includes the magnetic *g*- and *A*-tensors and the rotational diffusion tensor, R, which are expressed in a system of Cartesian axes fixed in the spin-labeled molecule. To reduce the number of parameters in the fittings and to simplify the simulation, the average rotational diffusion rate, *R*_bar_, was calculated by the fitting program using the relation *R*_bar_ = (*R*_per_^2^*R*_par_)^1/3^, where *R*_per_ is the perpendicular and *R*_par_ is the parallel component of the rotational diffusion [26]. *R*_bar_ was converted to the parameter rotational correlation time, τ_c_, following the relationship τ_c_ = 1/6 *R*_bar_. In this work, the spectra were simulated with a model of a single spectral component. Similar to previous studies [27,28], the magnetic parameters were determined based on a global analysis of the overall spectra obtained in this work, and all of the EPR spectra were simulated using the same predetermined parameters. Input parameters of tensors *g* and *A* were: *g_xx_* = 2.0082; *g_yy_* = 2.0060; *g_zz_* = 2.0022; *A_xx_* = 7.5; *A_yy_* = 7.0 G and *A_zz_* = 31.5 G.

### 3.7. Fluorimetric Detection of Mitochondrial-Derived O_2_^•−^

Mitochondrial production of superoxide anion was evaluated during the exposure of trypomastigotes to 1.5, 3.0, 6.0, 15.0 and 30.0 µM of (−)-elatol using the fluorescent O_2_^•−^ sensitive, mitochondrial-targeted probe MitoSOX [3,8-phenanthridinediamine, 5-(6-triphenylphosphoniumhexyl)-5,6-dihydro-6-phenyl]. Trypomastigotes (2 × 10^7^ cells/mL) were loaded with 5.0 µM MitoSOX for 10 min at room temperature (22 °C) and then washed with the KH (Krebs-Henseleit) buffer (pH 7.3) containing 15 mM NaHCO_3_, 5 mM KCl, 120 mM NaCl, 0.7 mM Na_2_HPO_4_ and 1.5 mM NaH_2_PO_4_ before the assays. Loaded cells were exposed to the stimuli, and after different times the fluorescence was measured in a fluorescence microplate reader (Victor X3, PerkinElmer) at λ_ex_ = 510 nm and λ_em_ = 580 nm. The oxMitoSOX becomes highly fluorescent upon binding to nucleic acids. In some of the experiments, cells were exposed to 10.0 µM AA, a stimulus known to induce O_2_^•−^ production by mitochondria [29].

### 3.8. Lipid Peroxidation Assay

Trypomastigote forms were incubated in the DMEM medium with 1.5, 3.0, 6.0, 15.0 and 30.0 µM of (−)-elatol for 3 h, at 37 °C. The extent of lipid peroxidation was determined as the amount of TBARS in terms of MDA. After incubation, samples (0.5 mg protein) were heated in a solution containing 0.37% thiobarbituric acid, 15% trichloroacetic acid, and 0.25 N HCl at 95 °C for 45 min. After cooling, the absorbance was read at 532 nm and the concentration of TBARS was calculated based on a ε value of 153,000 M^−1^cm^−1^ [30].

### 3.9. DNA Fragmentation

We analyzed DNA double-strand ruptures *in situ* by TUNEL (Terminal Deoxynucleotide Transferase dUTP Nick End Labeling). For this, trypomastigotes (1 × 10^7^ cells/mL) were treated with 1.5 and 3.0 µM of (−)-elatol for 24 h, after the cells were subjected to the TUNEL assay according to the manufacturer’s instructions. The compound actinomycin D 10.0 µg/mL was used as a positive control. The nuclei were counterstained with propidium iodide. Cells that have undergone DNA double-strand ruptures should fluorescence brightly, unlike the untreated cells. Fluorescence was observed in a fluorescence microscope Olympus BX51 (Olympus^®^) and pictures were captured with a UC30 camera (Olympus^®^). 

### 3.10. Cell Volume Determination

Trypomastigotes (1 × 10^7^ cells/mL) treated with 1.5 and 3.0 µM of (−)-elatol for 3 and 24 h, were collected by centrifugation, washed twice in PBS, resuspended in PBS and analyzed by fluorescence-activated cell sorting using a FACSCalibur flow cytometer. The compound actinomycin D 20.0 mM was used as a positive control. A total of 10,000 events were acquired in the region previously established as that corresponding to the parasites. Histograms and analysis were performed in CellQuest software; FSC-H represents the cell volume.

### 3.11. Evaluation of Autophagic Vacuoles

The autophagic vacuoles were evaluated using MDC labeling [31]. For this, trypomastigotes (1 × 10^7^ cells/mL) were treated with 1.5 and 3.0 µM of (−)-elatol for 24 h at 37 °C. Thus, the cells were incubated with 0.05 mM of MDC in PBS for 15 min at 37 °C. After incubation the cells were washed in PBS two times. MDC stain was analyzed by fluorescence microscope Olympus BX51 (Olympus^®^) and images were captured using a UC30 camera (Olympus^®^). In some experiments, cells were pre-treated with wortmannin, a potent PI3-kinase inhibitor, before induction of autophagy.

### 3.12. Statistical Analysis

The data shown in the graphs are expressed as means ± standard deviation of at least three independent experiments. Data were analyzed with one-way and two-way analysis of variance (ANOVA), significant differences among means were identified by Tukey post-test. *p* ≤ 0.05 was adopted as the minimum criterion of significance. Statistical analyses were performed using the Statistica™ software package.

## 4. Conclusions

**Figure 11:**
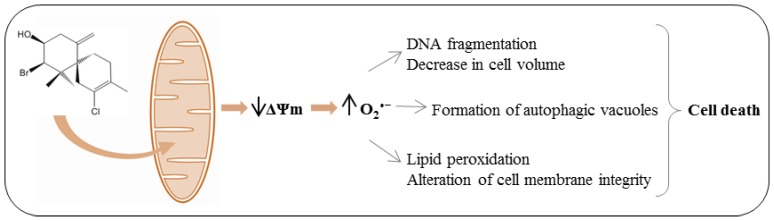
Mechanistic assumptions about the trypanocidal action of (−)-elatol. (−)-Elatol induces mitochondrial depolarization followed by an increase of ROS production through theelectron transport chain. This would affect all cell structures and function leading to cell death.

Our results indicate that the trypanocidal action of (−)-elatol is associated with mitochondrial depolarization followed by an increase of ROS production through the electron transport chain, which affects all cell structures, including mitochondria, leading to different types of parasite death (Figure 11). In this view, the mitochondria might be the initial target organelle of (−)-elatol. This hypothesis agrees with many other studies and strengthens the idea that mitochondria might be a target for trypanocidal action of new compounds [9,14,32,33]. On the other hand, we could also speculate that the initial event induced by (−)-elatol would be the increase of mitochondrial ROS, induced for example by a decrease in the antioxidant enzyme activity of the parasite. In this case, the mitochondrial dysfunction induced by (−)-elatol, described here, would be a consequence of the increase of ROS. Both situations are conceivable and are well supported by the “Reactive Oxygen Species (ROS)-induced ROS-release” (RIRR) process [34]. However, based on the small time course and also on the small concentrations of (−)-elatol in the Rh 123 assay results compared to mitoSOX assay results we strongly believe that the depolarization of mitochondrial membrane is the initial event undergone by the (−)-elatol-treated trypomastigotes (Figure 11).
